# How Do Spouses Experience Living Alone After Their Partner With Dementia Moves Into a Care Home?

**DOI:** 10.1111/jocn.17831

**Published:** 2025-06-02

**Authors:** Barbara Pope, Leslie Gelling, Sharon Holland, Chantel Cox

**Affiliations:** ^1^ Bournemouth University Fern Barrow Poole Dorset UK

**Keywords:** ambiguous loss, care homes, carer spouses/partners, grief, late onset dementia, loneliness

## Abstract

**Aims:**

This systematic review aims to explore spouses' lives after their partner with dementia moves to a care home facility. It will review existing peer‐reviewed papers written between 2002 and 2022 from English‐speaking parts of the world. It will investigate what is already established and underline where there are information gaps.

**Background:**

According to statistics, approximately 311,730 people with dementia currently reside in a care home. Many of these people will have a living spouse who will have to acclimatise to living alone and may experience anxiety and distress after this change.

**Design:**

A systematic search found that all the research papers met pre‐defined inclusion and exclusion criteria and were published between 2002 and 2022. Papers were identified and reviewed using the Critical Appraisal and Skills Programme (CASP) to evaluate the papers.

**Method:**

Databases searched included APA PsycINFO, MEDLINE Complete, Complementary Index, CINAHL Complete and Academic Search Ultimate Directory of Open Access. In total, 1390 papers were found; eight papers were identified; five were qualitative, and three were quantitative and analysed thematically. The Preferred Reporting Items for Systematic Reviews and Meta‐Analyses (PRISMA) checklist was used to support the presentation of this systematic review.

**Results:**

Detailed thematic analysis of the eight research studies included in this review identified three broad themes: (a) loss of a shared life, (b) visiting their partner in a care home and (c) grief, depression and ‘unable to move on’. These aspects have been shown to adversely impact the physical and mental health of the community‐dwelling spouse, which increases their exposure to depression.

**Conclusions:**

The selected papers showed persuasive evidence of the state of the community‐dwelling spouse's social, mental and physical health, which became a barrier to them moving forward with their lives. The needs of the community‐dwelling spouse have been under‐researched once their partner with dementia enters a care home. Further research is needed to understand how and when interventions should be offered to this group of people and which interventions might be most effective.

**Relevance to Clinical Practice:**

This research will help to disseminate clinical knowledge to nursing and other professionals, who will be able to appreciate the effect of moving a lifelong partner with dementia into a care home and be able to appreciate the uncertainties the community‐dwelling spouse feels at this time. With this information, they could identify spouses who are more vulnerable to the risk of not managing this phase of their lives and suggest appropriate support networks.

**Trial Registration:** This Systematic Review is registered in PROSPERO: 309784

**Summary**

- Improved spousal experiences of health and wellbeing.
- Prevention of depression, grief and loneliness.
- Empowering spouses to experience positive feelings during this phase of their lives.

## 1 Aims

This paper will present the findings of a systematic review undertaken to explore the experiences of spouses after their partner with dementia has moved into a care home. A systematic search identified eight research papers, which were then analysed to reveal three overarching them. This systematic review will present and discuss these themes, and recommendations will be made for practice and future research. ublb.

## 2 Background

According to the Dementia Statistics Hub, 982,000 it is estimated that there are 982,000 living with a diagnosis of dementia in the UK, and this is expected to rise to 1.4 million by 2040 (Carnell Farrar 2024). As people are living longer, the risk of developing dementia increases, becoming a challenging concern for older people and their carers alike (Livingston et al. 2017). Globally, 55 million people are living with dementia, with this number predicted to rise by 10 million every year, and it is the seventh leading cause of all deaths amongst all diseases (WHO 2023, and Alzheimer's Disease International London 2015) The impact of dementia is developing rapidly and is a societal crisis in many parts of the world.

As dementia progresses, most people with this condition will require assistance with their daily care needs (Eloniemi‐Sulkava et al. 2009). An increasing number of unpaid family members will engage in caring activities at home for a relative with dementia (Ballenger 2017). Recent figures suggest that approximately 700,000 family members are looking after a relative with dementia, with the majority (60%–70%) of these being women (Alzheimer's Research UK 2022). Although some people living with dementia can continue to live at home with family support until the end of their lives, many deteriorate, both physically and mentally and are not able to be cared for at home. Family carers might face issues which may result in a decision to move their relative with dementia into a care home. An estimated 311,730 people with dementia live in care homes (Alzheimer's Research UK 2022). Numbers are not collated for this group of spouses, and research in this area is limited. Consequently, spouses entering this phase of their lives are relatively unsupported by professionals; therefore, losing their partner emotionally and psychologically to dementia and further losing their physical presence in a care home may have a profound effect on spouses who might not have family or other social lines of support.

Dementia is not a disease but an overarching term used to describe a clinical syndrome triggered by multiple possible causes which affect the brain. Alzheimer's disease is by far the most common cause of dementia and accounts for 60%–70% of all dementia (Jalbert et al. 2008), and the second cause is structural damage resulting from vascular strokes, accounting for 20%–25% of dementias (Gale et al. 2018). Lewy Body dementias account for 4%–8% of dementia, and (Haider et al. 2025), there is an estimated 25% of all people with dementia with mixed dementia, that is, Alzheimer's and vascular dementia together (Custodio et al. 2017).

Dementia is progressive, unpredictable and non‐reversible. Symptoms vary from the early stages of the disease, with difficulty in concentrating, socialising problems and an inability to manage finances or complete simple tasks. The moderate stage of dementia sees further decline in abilities, such as significant memory deficiencies, needing help with activities of daily living, and communication problems. Late‐stage dementia usually requires wide‐ranging care and assistance for the loss of motor skills, incontinence and inability to eat (Reisberg et al. 1982).

People with dementia may become confused, have personality changes, display inappropriate behaviour, and have difficulty problem‐solving (WHO 2025). This may cause cognitive, sensory and psychological changes, problems with language and communication, and difficulty with problem‐solving and decision‐making. These symptoms can lead to an inability to perform simple actions of daily living (Eloniemi‐Sulkava et al. 2009). Due to its progressive nature, support with daily tasks may become inevitable (Lindeza et al. 2020). Family members providing care can impact their social and work commitments, and families may forego other routine activities to support each other.

The challenges of caring for people with dementia are considerable, often impacting the quality of life for the caregiver, which in turn may compromise the well‐being of the person living with dementia. Family members, particularly spouses, are considered the cornerstone of care provision (Potier et al. 2018), which can be problematic as older spousal carers are more likely to have their own physical or emotional health issues (Ask et al. 2014). Brodaty and Donkin (2009) identify the carer spouse as an ‘invisible patient’ vulnerable to physical and psychological burdens. Caring for a partner with dementia often leads to the spouse experiencing ‘carer burnout’ and exhaustion, which can subsequently lead to them being unable to meet the needs of their partner with dementia (Gaugler et al. 2010). Support for spousal carers in these circumstances may be inconsistent, as families may be unable to provide help, and financial constraints often limit social services input.

Research into informal spousal care of people with dementia whilst at home is well documented and has focussed on identifying and evaluating the negative symptoms of the physical and mental burden of care, depression and guilt (Brodaty and Donkin 2009; Hennings and Froggatt 2019; Crawford et al. 2015; Lloyd et al. 2014; Statz et al. 2021). Research has shown that caring for a spouse with dementia has a significantly higher level of burden and stress that may result in an adverse health outcome than non‐dementia caregivers (das Chagas Medeiros et al. 2000; Rees et al. 2001). The significant challenges of looking after a partner with dementia may lead to them moving into long‐term care. These challenges may be due to the complexities of dementia, which are giving cause for concern, for example, incontinence, wandering, disturbed sleep patterns or aggression. Conversely, there may be a deterioration in the carer spouse's health, meaning they can no longer provide the level of care needed (Gaugler et al. 2007; Crawford et al. 2015). The breakdown of care at home often means the spousal carer must acknowledge the inevitability of their long‐term partner with dementia moving into a care home.

In contrast to the burden and stress the spousal caregiver feels, Quinn et al. (2022) and Johansson et al. (2022) reported encouraging aspects of dementia caregiving, emphasising positive aspects of dementia care by spouses. Positive aspects of care included role fulfilment, pride in delivering high standards of care, and a sense of reciprocity, purpose and recognition of their marital relationship. The spouse considers they are the most suitable person to provide skilled care to their partner because they understand their needs and preferences better than anyone else.

The motives for transition to a care home are often complex. Furthermore, some spouses will have had this decision made for them by someone else, such as family members or social services, which may feel like a criticism of their abilities (Hennings and Froggatt 2019; Lord et al. 2016). This critique could impact the carer spouse's mental well‐being, as many will have been reluctant to give up their role as carers. Thoughts of being a failure or ‘not trying hard enough’ may add to feelings of guilt or remorse after their partner moves into a care home.

Attention is often focused on supporting the partner with dementia in the care home rather than the carer spouse (Kitwood and Bredin 1992; Egilstrod et al. 2019). Moving their partner with dementia into a care home might incur feelings of anxiety that professional carers will not meet their exacting standards of personalised care for their partner (Quinn et al. 2022). It is important to note that these positive and negative aspects of caregiving are not polar opposites and can exist together in varying degrees. In relinquishing the day‐to‐day care and management to care home staff, the spousal carer might experience a further change in identity from being a spouse providing twenty‐four‐hour care to that of visiting their partner in a care home.

Existing research examining family carers who have a family member with dementia in a care home usually refers to all family members, with spousal experiences included separately in the summary (Hennings and Froggatt 2019; Cottrell et al. 2020; Statz et al. 2021). However, evidence suggests that spouses are affected by different emotional and physical stressors than adult children experience (Hennings and Froggatt 2019; Schulz et al. 2004). This aspect has not been explored in sufficient depth.

Available literature on community‐dwelling spouses' adjustment to this new stage of their lives is scant (Førsund et al. 2015). This stage of life is referred to as ‘being in limbo’, as the ambiguous loss of a partner with dementia places the spousal carer in an indeterminate emotional state, which can cause unresolved stress and grief (Hemingway et al. 2016). Pauline Boss published a pivotal study on this liminal state in the 1970s (Boss and Yeats 2014) and coined the term ‘ambiguous loss’. Her work on spouses whose partner with dementia was physically ‘present’ but mentally unavailable described this phase of ambiguous loss. This is echoed by Rollins et al. (2008), who described this phase as ‘married widowhood.’ Having a partner who is ‘mentally lost’ to them means the remaining spouse is unable to move on with their lives and is ‘frozen’ in grief, which can lead to coping mechanisms becoming ‘blocked’. Spouses also reported that they could not begin to grieve or move forward until their partner with dementia had died. Thus, they were waiting for ‘closure’ or resolution of their liminal situation (Mullin et al. 2013; Førsund et al. 2015; Hemingway et al. 2016). With the community‐dwelling spouse's identity linked to their role of spouse/partner for a significant part of their adult life, living alone, neither widowed, divorced or single, can be a life‐altering adjustment to manage (Couture et al. 2020; Ahlström et al. 2021). Going forward in this liminal state can be challenging and negatively impact spouses.

Evidence suggests many spouses feel guilt by not abiding by their marriage vows or promises, whilst others feel relief at the loss of the physical and mental burden of care (Afram et al. 2015; Davis et al. 2019; Brooks et al. 2021). Some spouses can pick up the chapters of their lives before their caring role and re‐enter social settings (Mausbach et al. 2014). However, many spousal carers spend a portion of their day at the care home, playing a vital role in helping their partner to maintain their identity and provide information to staff to plan for the care of their spouse (Crawford et al. 2015).

Førsund et al. (2015), Hemingway et al. (2016) and Ahlström et al. (2021) suggest that transient feelings of ‘couplehood’ and ‘together but apart’ are common in spouses when one lives in a care home. Current evidence does not address the holistic and wide‐ranging breadth of experiences felt by community‐dwelling spouses, as much of the research focuses on specific topics such as visiting (Førsund et al. 2016), couplehood or health‐related quality of life (Bleijlevens et al. 2015; Førsund et al. 2015). These issues merit more detailed consideration to address current knowledge and understanding gaps.

## 3 Methods

Search terms were developed using the Population, Exposure and Outcome (PEO) method. A PEO tool helps to identify the question in qualitative research. The search was restricted to peer‐reviewed papers and grey literature between 01/01/2002 and 30/08/2022; all papers considered were in English. Non‐peer‐reviewed articles, editorials, reviews, conference reports and book chapters were examined to contribute to the search process but were not part of the systematic review. All findings were reported following the 2020 PRISMA checklist for systematic reviews (Page et al. 2021). Guidelines for reporting parallel group randomised trials (Supporting Information).

Patient/Population: Spouses who have moved their partner into a dementia facility.

Exposure: To loneliness, loss of partnership/identity. Relief of burden of care.

Outcome: Experiences, negative or positive, to this phase of life.

This review will seek to answer the above review question, which was informed by a Participant Exposure Outcome (PEO).

To synthesise and summarise existing knowledge and identify gaps in the literature over the last two decades.

## 4 Design

### 4.1 Search Strategy

A detailed and thorough search was conducted and guided by the Preferred Reporting of Items for Systematic Reviews and Meta‐Analyses (PRISMA) (Page 2021). The review protocol was registered on the International Prospective Register of Systematic Reviews.

### 4.2 Data Source

Electronic Bibliographical Databases Were Searched, Including:

APA PsycINFO, MEDLINE Complete, Complementary Index, CINAHL Complete, Academic Search Ultimate, Directory of Open Access Journals, SocINDEX, Full Text, SwePub, ScienceDirect, Supplemental Index, Education Source, APA PsycArticles. Boolean operators and truncation were used to connect search terms. Other hand searches via citations and Google were conducted, and experts in the field were contacted. A PROSPERO search identified no previous or recent reviews linked to this topic.

### 4.3 Study Selection

Keywords, synonyms and (MeSH) terms were used to identify the search papers (see Table 1) and all papers met the pre‐determined inclusion and exclusion criteria (see Table 2).

**TABLE 1:** Search terms.

Dementia OR Alzheimer* OR cognitive impairment OR memory loss OR vascular dementia
AND care home OR residential care OR care home OR residential home OR long‐term care
AND spous* OR partner OR wife OR wives OR husband OR couple OR couples OR domestic partners OR informal care OR carer burden
NOT end‐of‐life care OR palliative care OR death OR dying OR terminally ill
NOT care at home OR care in the community OR home care.

**TABLE 2:** sets out the inclusion/exclusion criteria and inclusion and exclusion criteria.

Inclusion criteria	Rationale for inclusion
Husband, wife, partner or same‐sex partner, caring for a spouse/partner with dementia who is now in a long‐term care home.	Spouses have a unique experience when their partner with dementia moves into a care home.
Married/partnership lasting for 20 years or more.	Long‐lasting relationships encompass more of the history between spouses/partners.
The carer spouses/partners must have been the primary carer.	The journey of caring for a spouse before moving to a care home is relevant because of their perceived role as a carer and their identity within the spousal dyad.
Any cause of late‐onset dementia	The type and stage of dementia are not relevant to this study as each person with dementia will have been affected differently.
People with dementia are diagnosed when they are > 65 years old.	Helpful information can be found through relevant dementia associations and grey literature.
Dates researched 2002–2022	To observe papers written over the past two decades and to establish how this subject has been investigated.
Papers written or translated into English.	Easy to comprehend in the researcher's language
Partner with dementia to have been in the home longer than 6 months	Allows for community‐dwelling spouses to have experienced living alone

Exclusion criteria	Rationale for exclusion
Early onset dementia	People with early onset dementia have different family and spousal obligations as they may be working and raising a family.
Adult child carers and other family members	Adult child carers have a different relationship with a parent with dementia and may have family commitments of their own.
Editorial and conference papers	There would not be enough robust information.
Papers not written in English.	The translation may not be exact.
End‐of‐life care	End‐of‐life care is a complicated grief system which has been widely researched.

The search terms can be seen in Table 1.

## 5 Methods

### 5.1 Critical Appraisal

Critical Appraisal Skills Programme (CASP 2022) tools were used to critically appraise each of the papers considered for inclusion in the review. Three of the eleven identified papers were not included in the review because they did not meet the full criteria. Any inconsistencies were settled by discussion or consensus with the supervisory team, and an independent reviewer was consulted if needed.

### 5.2 Screening Process

**FIGURE 1:**
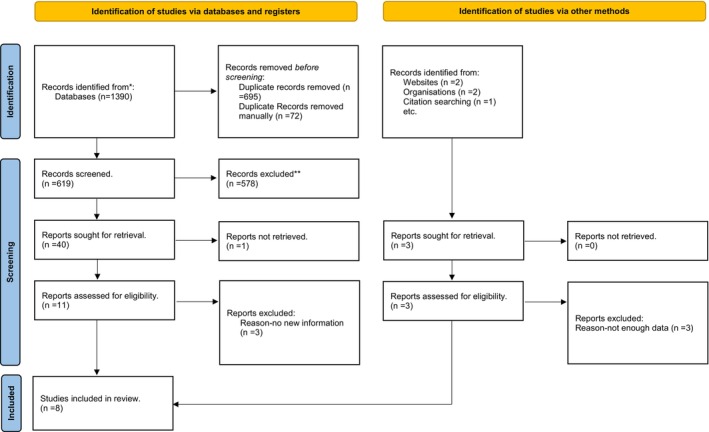
PRISMA flow chart adopted from Page et al. (2021). [Colour figure can be viewed at wileyonlinelibrary.com]

The Preferred Reporting Items for Systematic Review and Meta‐Analysis (Page et al. 2021) were used to ensure a transparent process of screening the papers. Screening of the papers was undertaken in two stages. The first stage was to review the title and abstract online against the inclusion and exclusion criteria. Papers at this stage were either included, excluded or filed as undecided. The undecided papers were then added to the included papers for full reading. Three independent academics independently reviewed 62 (10%) of the excluded papers to ensure the credibility of the review process. The second stage involved reading the whole paper against the inclusion/exclusion criteria; again, four (10%) excluded papers were reviewed independently. Tracking the references throughout the screening process was done to show transparency and provide an audit trail. Figure 1 shows the numbers involved and how they were reported. The Preferred Reporting Items for Systematic Reviews and Meta‐Analyses PRISMA (Page et al. 2021) were used to ensure the transparency of the screening process. Data research papers were managed using EndNote X9 bibliographic software. PRISMA diagram Table 3.

**TABLE 3:** Broad Themes Identified.

Feelings of loss	Loss of shared memories Loss of shared past life Loss of a future together
Visiting partner in the care home	Visiting as surveillance Visiting to maintain couplehood. Visiting to relieve loneliness. Visiting out of duty, loyalty and love
Grief, depression and unable to move on	The guilt felt by the community‐dwelling spouse at moving their partner into a care home. Guilt at trying to resume a ‘normal’ life. Depression was similar to before their partner moved into a care home. Guilt at feeling relieved of the burden and responsibility of care. Feeling ‘frozen’ and unable to move on.

### 5.3 Data Extraction

A data extraction tool was developed to collect relevant data from each paper selected for inclusion in the review. Key data is presented:

Who were the authors?

Were they sponsored? If so, by whom?

Were the aims of the study clearly stated?

Where and when did the study take place?

Duration of study.

Year of publication.

Were ethical issues addressed?

Methodology used in research.

How were participants selected?

Numbers and characteristics of participants.

Data collection methods.

Key themes identified.

Discussion of findings.

Limitations acknowledged.

Recognition of bias.

Recommendations for further research.

## 6 Results

Eight papers were included in the review. Five papers were qualitative studies, two by the same authors and three by separate authors. Two were from Sweden, one from Canada, one from Norway, and one from the UK. Three papers were quantitative studies, all three being from the USA. Three papers were rejected. One paper only had three spousal participants out of ten; the second paper evaluated a prognostic tool for the burden and depression of spouses after their partner moved into a care home, and the third paper was rejected because the data was extracted from a longitudinal study using a method which proved not to be sensitive to capture data.

Three broad themes emerged from the analysis. See Table 3.

1. Loss
  - Of a shared past life,
  - Loss of shared memories
  - Loss of a future with their partner.
2. Visiting their partner in a care home.
3. Grief, depression and inability to move on with their life.

These three themes provide important insights into the experience of a spouse living alone after their partner with dementia moves into a care home.

### 6.1 Loss

#### 6.1.1 Of a Shared Past Life

The feelings of a shared life are important to many couples, as sentiments of mutuality and enduring memories of their time together form a relationship which may have been gradually eroded by dementia. Førsund et al. (2015) draws attention to a loss of shared daily life, revealing feelings of being alone in their home, not part of a relationship and a sense of emptiness for the community‐dwelling spouse. One of the respondents in this study reported:

You think you have been together for so long, done things together, built a home together. And suddenly there you are. You are alone … (pause) Am I going to live in this big house where we somehow did … ? (Førsund et al. 2015, Participant 5).

Physical separation amplified this feeling of mental separation and loss, which was connected to the home and physical spaces once shared as a couple. One participant illustrated this sentiment by saying ‘empty chair, empty bed’ and describing his feelings for his wife as a ‘non‐presence’. Hemingway et al. (2016) further describes a participant saying, ‘When you place a person in a care facility, you lose them’. Another participant likened their situation to being ‘divorced’. Transition to a care home heralds the beginnings of a separate life for the community‐dwelling spouse, and the result of the loss of a shared lifetime was shown to be profound.

#### 6.1.2 Of Shared Memories

The loss of ability to reminisce or resolve divergence within the marriage dyad can cause a feeling of loss to the community‐dwelling spouse. The mutual history of both individuals involved in the relationship is now the sole responsibility of one person, as memories rest with the community‐dwelling spouse alone (Førsund et al. 2015). This feeling of being left alone with memories that once belonged to a couple generates a sense of isolation.

When a partner with dementia was shown photos or objects linked to family and home (Mullin et al. 2013), the result was often discouraging, as individuals with dementia are often unable to engage or acknowledge any of these memories or recognise them in a meaningful way. The community‐dwelling spouse felt the loss of being able to reminisce intensely. The cognitive deterioration caused by the dementia resulted in a lack of recognition, which helped to confirm a chasm in their dual identity as a couple. The notion of ‘I’ versus ‘We’ expressed how the community‐dwelling spouse felt about their new status (Førsund et al. 2015; Hemingway et al. (2016)). Many participants described their marriage vows as being the prime inspiration for their continued commitment to their partner with dementia. Some felt they were still married, and others felt alone but married, though this was not a fixed position. In Hemingway et al.'s (2016) paper, she notes one wife saying:

So basically, it is like a divorce, a person, I mean he does not remember me, so, he just remembers the people who are there. Can you see the hurt? Can you see the pain?. (Hemmingway 2016)

When dementia interrupts memory processes, collaborative reminiscing may be lost, resulting in disjointedness within the dyad.

#### 6.1.3 Loss of a Future With Their Partner

The loss of a shared future together affected the community‐dwelling spouse as plans for a joint future gradually evaporated with the progression of dementia (Førsund et al. 2015). The community‐dwelling spouse's life may become ‘frozen’ and the future increasingly unknown. Plans made together can no longer be fulfilled. This sense of losing their future also resulted in insecurity (Førsund et al. 2015). The difficulty of not knowing how long they would retain their partner as a meaningful part of their life or what would happen to them if the community‐dwelling spouse died first was of immense concern to the caregiver spouse. Hemingway et al. (2016) describes how spousal caregivers felt they now had to learn new tasks and were solely responsible for everyday duties, such as running the house and other responsibilities which used to be performed by the person living in a care home with dementia. It was not the need to undertake additional tasks that caused added anxiety. Instead, these responsibilities reminded them that their partner performed them within their relationship.

The loss of couplehood felt by the spouse who is left to live alone after their partner with dementia moves into a care home can be overwhelming. Spouses described how they continue to keep their home and garden in the same state after their partner moves into the care home, as this embodies their mutual life prior to their partner's dementia (Hogsnes et al. 2013). Though it was accepted that many emotional, practical and physical losses might have occurred since a diagnosis of dementia, Førsund et al. (2015) suggests that couplehood further disintegrated when one spouse lived alone and visited their partner in a care home. Shared spaces within the home and garden assume an altered significance, to which the community‐dwelling spouse must adapt.

The inability to recall happier times, intimacy, shared hobbies, communication and events together added to the loneliness and sadness the community‐dwelling spouse felt. Hemingway (2016) describes how spouses felt once they realised their previous life had evaporated. Spouses said of their newfound position in life:

It is just that you have to learn to be on your own, you know. I think that the hardest thing is that you have a husband, but you have nothing.

The loss of a joint future was also keenly felt. The future was uncertain, unknown and unanticipated. Their identity as a married couple had been fractured, and only the community‐dwelling spouse was aware of this as the dementia of their partner deteriorated.

### 6.2 Visiting Their Partner in a Care Home

Transition to a care home can cause distressing outcomes for the caregiver spouse. In a spousal partnership, the carer spouse often makes modifications in daily life for their partner with dementia, which disguises the extent of their dementia (Hemingway et al. 2016). When their partner with dementia enters a care home, the actual degree of dementia becomes evident, which is perceived by the community‐dwelling spouse and their family as a worsening of their dementia, leading to further guilt at having placed them into a care home.

Visiting their partner in the care home became necessary for several reasons. Firstly, spouses felt the need to continue their marriage and preserve communication. Visiting gave a purpose for the marriage as being together, confirming their identity as a couple (Førsund et al. 2016). Attempts at communication became frustrating to the community‐dwelling spouse as dementia progressed; nevertheless, many continued to persevere (Hogsnes et al. 2013). Secondly, some spouses visited as surveillance to ensure their partner was being looked after adequately. This also led to spouses providing selected care, such as assisting with eating, taking them out for a walk or a car ride, and leaving the staff to provide the intimate care their partner needed.

Perceived disappointment regarding levels of care was notable and led to anxiety for the community‐dwelling spouse (Mullin et al. 2013). Instructions regarding the care of their partner with dementia given to staff were sometimes not adhered to; therefore, expectations of care by the carer spouse and the apparent delivery of care from staff did not match, leading to increased visits by the spouse for surveillance purposes. Mullin et al. (2013) describes this as ‘visiting as surveillance’. Gaugler et al. (2010) stated that spouses felt ‘compelled’ to remain involved in caring for their partner in the care home for the same reason. Visiting as a means of supervision, to ensure their partner was receiving acceptable care, may be one of the reasons for visiting. Mullin et al.'s (2013) paper describes how one husband said:

I want to be here (in the nursing home) to convince myself that erm (.) she's (.) everything's OK. (Ben) (Mullin et al. 2013)

Thus, the frequency of visits becomes determined by a dual function, either to help the staff in caring tasks or to maintain surveillance.

Visits by the community‐dwelling spouse were also for their fulfilment. Many missed the company they once had and visited to alleviate their loneliness (Førsund et al. 2016). Feelings of guilt likewise accounted for visits, believing they should be there and not doing other social activities with their ‘free time’. One of the participants in Hogsnes's (2013) paper said:

Things are a little easier now, of course, but I don't know…I can't seem to stop talking about it. If I'm somewhere out on a boat, for example, I'll just start talking about how I really should have gone for a visit instead. (IN 6)

Many spouses spoke of their sadness and guilt when they had to leave the home (Hemingway et al. 2016), saying staff helped with their exiting by diverting the person with dementia to another activity.

Førsund et al. (2016) found that many community‐dwelling spouses visited their partner with dementia to alleviate their loneliness and forge a new brand of couplehood. Love and devotion, a committed relationship and their values of marriage supported this undertaking. Spouses' visits were arranged to coordinate with their partner's moods and activities, for instance, when the partner was more likely to be awake and receptive to them (Førsund et al. 2016). Visiting was organised at specific times, for example, in the mornings when their partner was more likely to be responsive or at supper time to alleviate sundowning symptoms. One participant said:

I have to be there in the morning. Because he is very tired in the afternoon and then he gets so angry. I found out it is better when I visit in the morning. (Førsund et al. 2016)

Visiting may also be a continuation of care, providing limited personal care and guiding care staff towards a more personal level of care for their partner with dementia. Some spouses visited out of duty and loyalty to the person they share their marriage with (Hemingway et al. 2016), describing reciprocity of care as part of their marriage vows. Spouses reported that they felt guilty because their wedding vows promised to take care of each other ‘in sickness and in health’, and this is frequently felt like a broken oath, notably emphasised when ending their visits (Schulz et al. 2004; Gaugler et al. 2010). This is endorsed by Førsund et al. (2016), who found that saying goodbye at the end of a visit triggered distress and a feeling of abandoning their partner. One carer spouse noted how they felt guilt‐ridden because they had to ‘sneak away’ to avoid causing distress to their partner. Førsund et al. (2016) refers to a conversation between the interviewer and participant:

Interviewer—So you basically have to sneak away? Otto—yes, when she sits down and starts to think about the food, then I can sneak out. If I say “I will come back on Sunday” then it usually turns out OK. (sobbing) (Førsund et al. 2016).

However, Mullin et al. (2013) observed that as dementia progressed in their partner and communication and memory diminished, the visiting spouse's feelings of guilt decreased. As communication with their partner with dementia diminished, the structure and frequency of the visits declined. Guilt can also be part of the visiting arrangement, as spouses may feel they should visit their partner instead of doing other activities. One of the participants in *Førsund's* paper said:

I visit him two times a week. I think that is sufficient. He doesn't know who I am, or if I come there or not. It's a familiar face, a familiar voice. There is nothing more. (Førsund et al.' 2016)

Therefore, choices around visiting their partner in the care home are shaped by many aspects, such as supervision of care, alleviating their loneliness and guilt and maintaining couplehood. Thus, the frequency of visits becomes determined by a dual function, either to help the staff in caring tasks or to maintain surveillance.

### 6.3 Grief, Depression and Moving on

A longitudinal study from 1996 to 2000 by Schulz et al. (2004) concluded that the transition to a care home for a partner with dementia was challenging for the community‐dwelling spouse, as their depressive symptoms and anxiety were often as high post‐placement as they were when they were homecare givers. However, this conflicts with the evidence which suggests that the community‐dwelling spouse's depressive symptoms improved after their partner moved into a care home because of an increase in personal mastery and independence gained after placement (Khalaila and Cohen 2016; Mausbach et al. 2014). This concurs with Aneshensel et al. (2000), who cited some spouses who felt their lives were easier in practical terms after their partner went into a care home because they no longer provided physical care. These dichotomous findings are illustrative of unique long‐term partnerships, as the complex issues of depressive symptoms, anxiety and mastery are dependent on the distinctive and multifaceted experiences of being in a relationship.

Resuming a social life also presented dilemmas, as some community‐dwelling spouses felt like the ‘odd one out’ when invited to gatherings where there were couples (Hogsnes et al. 2013). They felt guilty and disloyal by enjoying themselves, feeling they should spend time with their partner instead. This element was also reported by some spouses who felt they were still so immersed in their situation that they could not participate in social settings and avoided them, adding that being widowed would have more explicit boundaries and expectations of how people would react to them (Førsund et al. 2015). This curtailed their social horizons after their partner transitioned to a care home, adding to an increased feeling of isolation. Hemingway (2014) described one spouse who said of their newfound position in life:

This is the way it is going to be and it is not going to get any better. I have got to do it myself now. (Hemmingway 2016)

The move into a long‐term care home means acknowledging that their partner with dementia will not be able to return home and resume their life with them; therefore, the community‐dwelling spouse may feel an acute sense of separation, loneliness and isolation. This liminal state can trigger feelings of anxiety, grief and depression.

However, moving on with life after their partner with dementia moved into care was difficult for some spouses, though not all encountered this impediment. Boss's (2009) work on the liminal status of ambiguous loss discussed the subject of mastery, for example, adaptation and coping strategies, versus resistance, which led to their ability to cope with their partner's dementia. In recent years, approaches have attempted to explore the predicament of the spouse living in this liminal construct. The paradox of this type of loss without physical death (Hemingway et al. 2016) intensifies the fragility felt by the community‐dwelling spouse. Spouses may feel a mixture of love, anger, resentment and guilt because of the effect this disease has had in reshaping both of their lives.

Less consideration is paid to this unique type of ambiguous loss, which can overwhelm people and can last for many years while their partner with dementia remains in the care home. Unlike death, there are no rituals, no closure and no change in identity from married to being a widow, leaving them to manage alone, lacking the customary support to move ahead with their lives. Additionally, this leads to coping mechanisms becoming ‘blocked’, which complicates the grief process and puts their life ‘on hold’ (Boss 2009).

However, guilt also emerged when the community‐dwelling spouse regained freedom from their caregiving tasks and began to move ahead with their altered circumstances (Hogsnes et al. 2013; Mullin et al. 2013). Some spouses felt their difficulties would end when their partner with dementia died, enabling them to reconstruct their lives without their partner (Mullin et al. 2013). They felt this would be easier than experiencing the daily losses brought about by watching their partner with dementia diminish, called ‘non‐death related loss’ by Hemingway et al. (2016). This is supported by one participant who reported feeling ‘frozen’ in their life and was waiting ‘till it was all over’ so she could start a new life (Førsund et al. 2016). However, guilt at having such thoughts compounds these feelings.

The concept that this liminal status and facing an uncertain future alone caused paradoxical feelings of hope and despair simultaneously was noted by both Førsund et al. (2016) and Hogsnes et al. (2013). Spouses in papers by Mullin et al. (2013) Førsund et al. (2016) and Hemingway et al. (2016) cited that they could not begin to grieve or move forward until their partner with dementia had died; thus, they were waiting for ‘closure’ or resolution of their liminal situation. One participant said:

This is what I'm saying… It's worse than death, goddamit… no doubt about it. It is, because if someone dies, you can start adjusting to the loss from that date. (IN 2) Førsund et al. (2016)

Because they still had a living partner, their status of ‘being married’ held them back emotionally from pursuing a new life or reconnecting with their old one of friendships and hobbies. Feelings of guilt that they should not be enjoying themselves or they should be with their partner in the care home led to further isolation, loneliness and even depression (Hogsnes et al. 2013). This ‘open‐ended’ loss may continue for many months or even years; thus, they cannot resume their life. This challenges the community‐dwelling spouse's identity (Førsund 2015). Changes in identity meant the community‐dwelling spouse had to perform a new and often unwelcome role of living alone yet married.

## 7 Discussion

This review aimed to discover how community‐dwelling spouses experience their lives once their partner with dementia has moved into a care home. Using qualitative research to investigate emotions and feelings has established the ‘lived experience’ of a person who has encountered a specific phenomenon (Braun and Clarke 2006; Smith et al. 2021). Using qualitative methodologies to explore the lives of community‐dwelling spouses is valid to elicit a deep understanding of their lives and draw attention to the issues faced once their partner has entered a care home. Data from quantitative papers seek out measurable topics, such as levels of depression, anxiety or visiting, and show a convergence between the lived experiences found through qualitative methodologies. The selected qualitative and quantitative papers would appear to concur that caring for the community‐dwelling spouse deserves further attention, including further research, better preparation for transitioning a partner to a care home and appropriate support after the partner enters the care home.

Predictably, most papers exploring spousal care of partners with dementia have concentrated on care within the home setting (Kitwood and Bredin 1992). Once a family member with dementia has moved into a care home, many papers conflate adult child carers and spousal experiences, which do not address the disparity between the needs and experiences of the community‐dwelling spouse and other family carers. As outlined in the Social Care Act 2014 Ch.23 (10), local authorities are now required to assess and promote the carer's well‐being. However, this relates to care at home. There has been some degree of improvement in the status of carers and their requirements in caring for some of the most vulnerable people in society at home. However, a review of current evidence suggests that there has been scant investigation into the liminal experiences of the community‐dwelling spouse once their partner with dementia has entered a care home. Carer spouses are often missed in the equation as the focus is given to the person with dementia. The potential physical and emotional impact on the individual residing alone when their partner with dementia has moved into a care home has been underestimated and under‐researched. This critical literature review has focussed on the consequences for the community‐dwelling spouse and identified important areas for future research.

Limitations in the selected papers.

Based on the selected papers, it is important to note that the impact of dementia was dependent on the nature of the marriage beforehand; therefore, the chosen papers in this review have limitations in their studies. None of the papers found during the search or selected for this systematic review interviewed spouses who stated they were ambivalent or unhappy in their marriage. Therefore, this group of people has been hidden from research.

Also absent from the research were the voices of different ethnicities, though it is acknowledged that many families of diverse cultures do not use care homes but look after their older generation at home. Additionally, same‐sex relationships and second marriages and partnerships were often overlooked. Not all selected papers investigated the gender of the caregiver, despite approximately 41% of men accounting for caring for a family member with dementia (McDonnell and Ryan 2013), though this proportion does not explicitly relate to spousal caregivers.

Additionally, it is important to note that the papers selected were from countries with different social care input levels to support families looking after a relative with dementia at home. This means there might be inconsistency between a country's provision for care home places and the frailty of the person with dementia when they enter care, reflecting on the support the spouses may have had. Although Schulz et al. (2004) briefly mentions financial constraints as a contributory factor to causing depressive symptoms, other papers did not discuss the impact of monetary issues. Nevertheless, the financial burden of moving a partner to a care home could lead to another obligation for the community‐dwelling spouse, leading to further unforeseen anxiety.

## 8 Conclusion

This literature review has provided a brief synopsis of eight papers from a global search on how community‐dwelling spouses experience their lives after their partner with dementia enters a care home. Papers provide compelling evidence of loss, depression, anxiety and grief, which become a barrier to moving forward with their lives. This stage of life seems unsupported for them, leaving them to live in an ambiguous state. The selected papers expose the research gaps, and further investigation needs to be undertaken to discover these groups of people. Notwithstanding these limitations, the papers reveal the nature of older spousal carers when caring for their partner with dementia and their life as a lone community‐dwelling spouse.

The most pertinent finding from the literature available is that more research into the lives of the community‐dwelling spouses is needed. The evidence from the selected papers has important implications for ensuring appropriate services and support systems are in place for spouses before, during and after moving their partner into a care home. Overall, these studies consistently highlight the complexity of the unique relationship between spousal partnerships after one of them with dementia enters a care home.

## 9 Relevance to Clinical Practice

Research has shown that adverse effects on the carer spouse's health and mental well‐being after their partner with dementia has moved into long‐term care remain for long periods. Studies have exposed this ambiguous period as a source of uncertainty, which causes incalculable stress and anxiety, leading to as much physical and mental distress as prior to their partner's move. The subsequent gains of relinquishing the delivery of physical care and supervision are matched with many losses accrued by learning how to live alone. By professionals being able to recognise this dilemma, the effects of the carer spouse becoming a ‘second patient’ to physical and psychological problems can be minimised. This paper will inform the clinical community that they can direct the community‐dwelling spouse to the appropriate groups and organisations that can help.

## Conflicts of Interest

The authors declare no conflicts of interest.

## Supporting information


Data S1.

## Data Availability

The data that support the findings of this study are openly available in BURO at BORDaR.
